# Highly Enhanced Photoreductive Degradation of Polybromodiphenyl Ethers with g-C_3_N_4_/TiO_2_ under Visible Light Irradiation

**DOI:** 10.3390/nano7040076

**Published:** 2017-04-03

**Authors:** Weidong Ye, Yingying Shao, Xuefeng Hu, Chulin Liu, Chunyan Sun

**Affiliations:** 1 Department of Chemistry, Shaoxing University, Shaoxing 312000, Zhejiang, China; zjpjyeweidong@163.com (W.Y.); hmilyariel@icloud.com (Y.S.); liuchunlin@usx.edu.cn (C.L.); 2 Key Laboratory of Coastal Zone Envirenmental Process and Ecological Remediation, Yantai Institute of Coastal Zone Research, Chinese Academy of Sciences, Yantai 264003, Shandong, China; xfhu@yic.ac.cn

**Keywords:** g-C_3_N_4_, TiO_2_, photoreductive, polybromodiphenyl ethers, visible light

## Abstract

A series of high activity photocatalysts g-C_3_N_4_-TiO_2_ were synthesized by simple one-pot thermal transformation method and characterized by transmission electron microscopy (TEM), scanning electron microscopy (SEM), X-ray diffraction (XRD), X-ray photoelectron spectroscopy, Brunauer–Emmett–Teller (BET) surface area, and ultraviolet–visible diffuse reflectance spectroscopy (UV-Vis-DRS). The g-C_3_N_4_-TiO_2_ samples show highly improved photoreductive capability for the degradation of polybromodiphenyl ethers compared with g-C_3_N_4_ under visible light irradiation. Among all the hybrids, 0.02-C_3_N_4_-TiO_2_ with 2 wt % g-C_3_N_4_ loaded shows the highest reaction rate, which is 15 times as high as that in bare g-C_3_N_4_. The well_-_matched band gaps in heterojunction g-C_3_N_4_-TiO_2_ not only strengthen the absorption intensity, but also show more effective charge carrier separation, which results in the highly enhanced photoreductive performance under visible light irradiation. The trapping experiments show that holetrapping agents largely affect the reaction rate. The rate of electron accumulation in the conductive band is the rate-determining step in the degradation reaction. A possible photoreductive mechanism has been proposed.

## 1. Introduction

Persistent organic pollutants (POPs) are of significant concern because they are bioaccumulative and harmful to human health [1,2,3,4]. Photocatalysis is one of the most effective technologies for the remediation of POPs [5]. TiO_2_ has attracted much attention in this field for the degradation of POPs due to its excellent photocatalytic capability, high chemical stability, and environmental friendliness [6]. However, TiO_2_ is only excited by UV light, and photoinduced hole–electron pairs can be fast recombined. Therefore, many efforts have been done to overcome these disadvantages [7].

Recently, carbon nitride (C_3_N_4_) with graphite-like structure—being a wide-band-gap semiconductor—has received wide attention in catalytic applications due to its high chemical stability and appealing electronic structure [8]. The optical band gap of g-C_3_N_4_ is 2.7 eV (*E*_CB_ = −1.3 V, *E*_VB_ = 1.4 V vs. *NHE*, pH = 7; CB: conduction band, VB: valence band, *NHE*: normal hydrogen electrode), which gives it promising performance in photocatalytic activity [9]. For example, g-C_3_N_4_ was able to split water into hydrogen or oxygen in the presence of an electron donor or acceptor under irradiation [10]. H_2_O_2_ could be activated by g-C_3_N_4_ for the oxidation of benzene to phenol [11]. g-C_3_N_4_ also showed good photocatalytic activities for the oxidation of various organic dyes under visible light irradiation [12]. Nevertheless, the separation of charge in the excited g-C_3_N_4_ needs to be improved to enhance the photocatalytic efficiency.

The synthesis of a heterojunction photocatalyst is a feasible way to overcome the disadvantages of TiO_2_ and C_3_N_4_ [13,14]. A heterojunction with proper band gaps can not only superpose the light response of composed semiconductors, but also help the transformation of photogenerated holes and electrons, and finally improve the photocatalytic capability [15]. Several studies have reported that the photocatalyst g-C_3_N_4_-TiO_2_ performed improved photocatalytic activity. For example, g-C_3_N_4_-TiO_2_ prepared by simple grinded could have better performance in the hydrogen evolution [16]. Hu et al. reported that the hybrid photocatalyst of g-C_3_N_4_-TiO_2_ showed enhanced photogenerated charge separation for dye degradation [17]. Therefore, g-C_3_N_4_-TiO_2_ can perform not only visible light response but also higher effective charge separation [18,19]. However, most of the studies have focused on the photocatalytic oxidation of g-C_3_N_4_-TiO_2_, while the photoreductive capability of g-C_3_N_4_-TiO_2_ for the degradation of pollutants has been largely neglected [16].

In this work, we prepared the heterojunction catalyst g-C_3_N_4_-TiO_2_ with simple one-pot thermal transformation. Decabromodiphenyl ether (BDE209) (Figure S1) is a typical persistent organic pollutant and was selected as the target substrate. The photoreductive activity of g-C_3_N_4_-TiO_2_ was performed by the degradation of BDE209. The g-C_3_N_4_-TiO_2_ showed highly enhanced photoreductive capability compared with g-C_3_N_4_. Trapping experiments were performed to further understand the rate-determining step of the photoreductive reaction and the reaction mechanism. To the best of our knowledge, it is the first time the highly-improved photoreductive ability of g-C_3_N_4_-TiO_2_ in the degradation of polybromodiphenyl ethers PBDEss under visible light irradiation has been researched.

## 2. Results

### 2.1. Catalyst Characterization

#### 2.1.1. TEM and SEM

**Figure 1:**
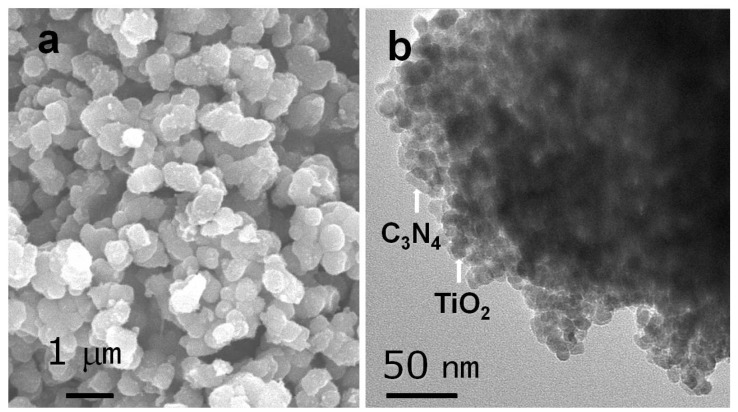
(**a**) Scanning electron microscopy (SEM) image of 0.02-C_3_N_4_-TiO_2_; (**b**) transmission electron microscopy (TEM) image of 0.02-C_3_N_4_-TiO_2_.

As shown in Figure S2 and Figure 1, the morphology and microstructure of g-C_3_N_4_ and g-C_3_N_4_-TiO_2_ were characterized by SEM and TEM. The SEM image of the sample shows small roundish particles, which have slightly irregular edges. The particle sizes are 0.2–0.5 μm (Figure 1a). The TEM image displays the inner structure of the catalyst. The TEM image of g-C_3_N_4_-TiO_2_ shows that TiO_2_ particles disperse on the surface of g-C_3_N_4_ with sizes 5–10 nm (Figure 1b). In the TEM image, roundish TiO_2_ particles also show the firm connection with the surface of the lamellar g-C_3_N_4_ to form the heterojunction interface, which contribute to the better transfer of photoelectrons and separation of charge carriers between g-C_3_N_4_ and TiO_2_. 

#### 2.1.2. XRD

**Figure 2:**
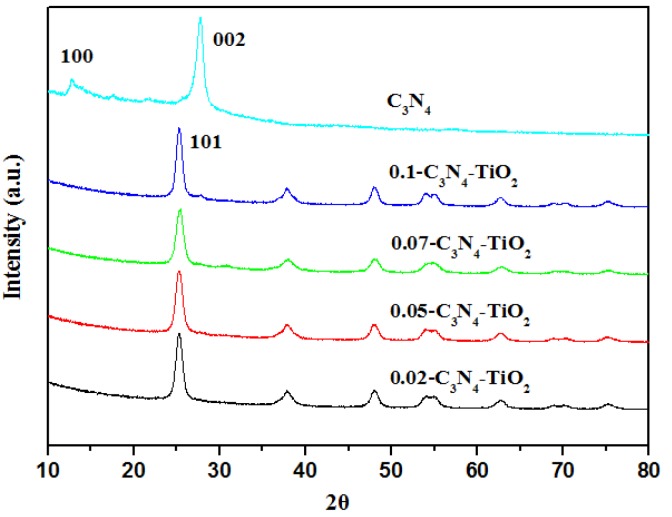
X-ray diffraction (XRD) pattern of g-C_3_N_4_-TiO_2_.

Figure 2 shows the X-ray diffraction patterns of X-C_3_N_4_-TiO_2_ and g-C_3_N_4_. The pattern of the g-C_3_N_4_ sample has two peaks. The small-angle peak (100) at 12.77 indicates the in-plane structural packing motif. The strongest peak at 27.62 is the characteristic interlayer stacking peak of the aromatic system, indexed as the (002) peak for graphitic materials [20]. The diffraction peaks of TiO_2_ exhibit the typical structure of anatase phase. When g-C_3_N_4_ is combined with TiO_2_, all hybrids of g-C_3_N_4_-TiO_2_ show the typical anatase TiO_2_ phase [17]. The characteristic peaks of g-C_3_N_4_ are not observed due to the low content in hybrids and relatively weak diffraction intensity of g-C_3_N_4_.

#### 2.1.3. X-ray Photoelectron Spectroscopy (XPS)

XPS measurements demonstrate the oxidation state and surface chemical compositions of 0.02-C_3_N_4_-TiO_2_. As shown in Figure S3, the C1s XPS spectrum shows three main peaks at 284.4 eV, 285.5 eV, and 288.2 eV, corresponding to the adventitious sp^2^ carbon atoms bonded to N inside aromatic units, C-(N)3 group. The XPS of N1s has three signals at 398.5 eV, 399.5 eV, and 401.2 eV originating from sp^2^ bonded N in the triazine rings, tertiary N in N-(C)_3_ and N in the amind group. Ti2p has two peaks corresponding to Ti2P_3/2_ at 458.6 eV and Ti2P_1/2_ at 464.3 eV. O1s shows two peaks at 529.8 eV and 531.4 eV, attributed to the O atom in O–Ti and surface OH, respectively.

#### 2.1.4. UV-Vis Absorption Spectra

**Figure 3:**
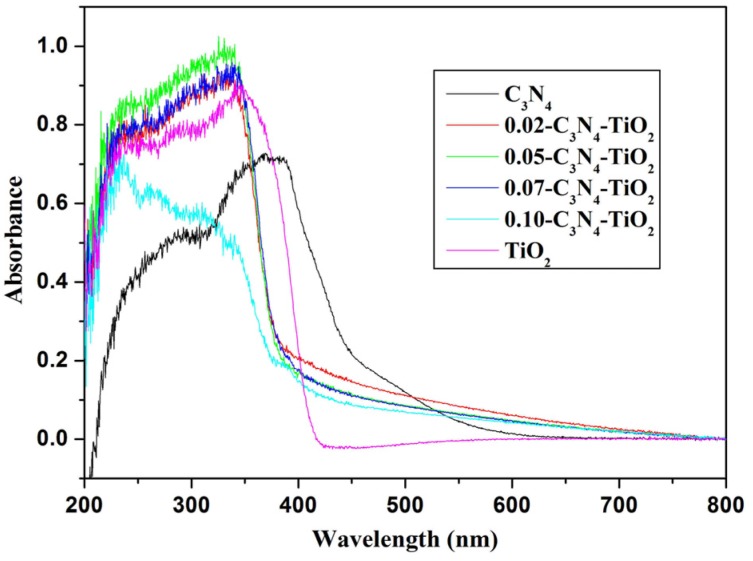
UV-Vis absorption spectra of g-C_3_N_4_-TiO_2_.

The optical properties of g-C_3_N_4_-TiO_2_ samples are investigated by UV-Vis diffuse reflectance spectra. As shown in Figure 3, without modifying the TiO_2_ particles, g-C_3_N_4_ shows broad absorption in the region of 215–600 nm, and the maximum absorbance appeares at 370 nm. TiO_2_ only shows absorption in the UV region. When g-C_3_N_4_ is combined with TiO_2_, all hybrids of g-C_3_N_4_-TiO_2_ show enhanced absorption in both UV and visible light regions. Obviously, the visible absorption of *x*-g-C_3_N_4_-TiO_2_ above 400 nm is the contribution of g-C_3_N_4_, which has a strong and wide absorption band in the visible region. It also implies that *x*-g-C_3_N_4_-TiO_2_ may have greater photocatalytic activity than g-C_3_N_4_ and TiO_2_. Moreover, slight changes could be observed with increasing TiO_2_ content, and among all hybrid samples, 0.02-C_3_N_4_-TiO_2_ showed relatively strong absorption intensity in the visible light region.

### 2.2. Photocatalytic Reductive Performance Study

#### 2.2.1. Degradation Kinetics

**Figure 4:**
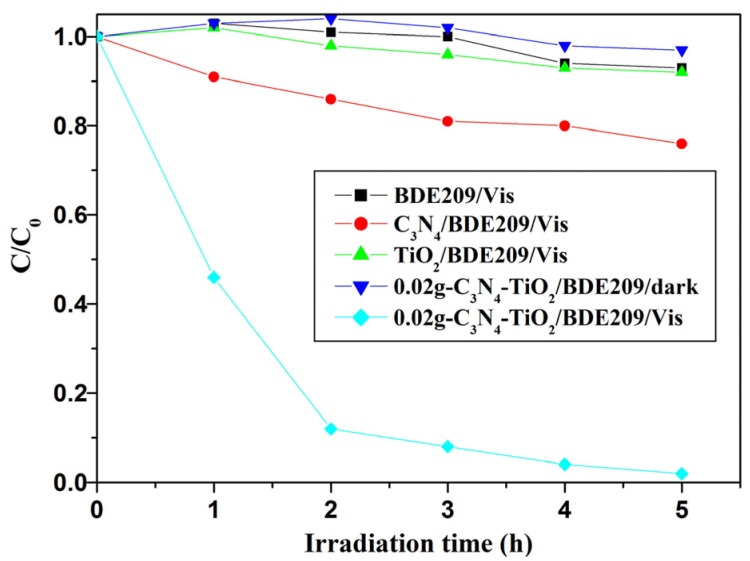
Temporal curves of the degradation of BDE209 under different conditions. BDE209: 1.0 × 10^−5^ mol/L; 0.02-g-C_3_N_4_-TiO_2_: 1 mg/mL; solvent: CH_3_OH; wavelength >420 nm; anoxic condition.

As seen from Figure 4, BDE209 itself shows no degradation under visible light irradiation. This means that BDE209 cannot direct photolysis in this condition. BDE209 also exhibits little degradation in the presence of TiO_2_, which has no absorption in visible light. In g-C_3_N_4_-TiO_2_/dark system, BDE209 cannot degrade. This means that the degradation of BDE209 cannot occur without irradiation. In the presence of g-C_3_N_4_ under visible light irradiation, BDE209 shows slightly degradation. However, rapid degradation of BDE209 is observed with 0.02-g-C_3_N_4_-TiO_2_ under visible light irradiation. More than 90% of BDE209 disappeared after 2 h of irradiation. The kinetics are fitted by pseudo-first-order process, giving a rate constant of 0.78 ± 0.02 h^−1^ (*t*_1/2_ = 0.89 h). Compared with g-C_3_N_4_ (0.052 ± 0.02 h^−1^), the kinetics rate improves 15-times.

The product analysis by gas chromatography paired with a microcell electron capture detector (GC-μECD) shows that the degradation of BDE209 by g-C_3_N_4_-TiO_2_ leads to the formation of its lower brominated congeners in a stepwise way (Figure S4). Before the irradiation, the only dominant GC peak is from BDE209. After 1 h irradiation, nona-BDEs appeares as main intermediates. After irradiation of 3 h, the octa-BDEs and hepta-BDEs are measured as the dominant intermediates. Hexa-BDEs and penta-BDEs are observed as main intermediates after 10 h irradiation. Hexa-BDEs and penta-BDEs gradually vanish, and transform to the lower intermediates at further prolonged irradiation to 24 h.

**Figure 5:**
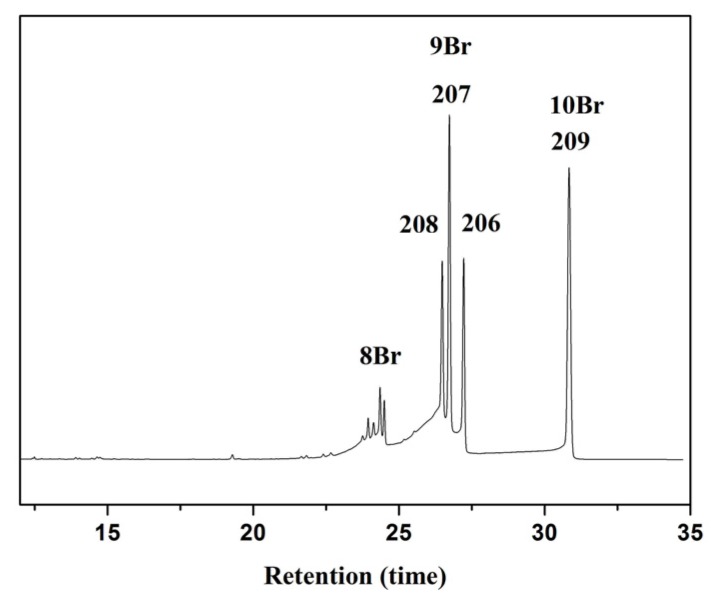
GC-μECD (gas chromatography paired with a microcell electron capture detector) chromatograms of degradation products of BDE209 by 0.02-g-C_3_N_4_-TiO_2_ in 2 h irradiation.

The first debromination step is the formation of nona-BDEs by losing one bromine atom, which provides direct information regarding the debromination pathway of BDE209. There are three nona-BDEs, named BDE206 BDE207, and BDE208, corresponding to *ortho*-, *meta*-, and *para*-debrominated intermediates of BDE209, respectively. As seen from Figure 5, all nona-BDEs are observed, identified to be BDE208, 207, and 206, respectively, according to their well-established GC elution times [3,4,6]. The relatively largest peak areas of nona-BDEs named BDE207 appears, indicating that the *meta* debromination is much easier than those from other positions. This is unlike that in TiO_2_ systems [6], in which the predominant degradation product of nona-BDEs is BDE206, but is similar to that in the g-C_3_N_4_ system [20]. This implies that the debromination patterns of BDE209 by photocatalytic degradation varies in the different systems. In the C_3_N_4_-TiO_2_ system, BDE209 preferentially adsorbs on the surface of g-C_3_N_4._ Therefore, the debromination pathway is not only affected by the bond dissociation energy of C-Br but also restricted by the space position of BDE209 and photocatalyst, which results in the change of the debromination patterns of BDE209. It also indicates that the reductive debromination of BDE209 occurs on the surface of C_3_N_4_-TiO_2_.

#### 2.2.2. The Effects of Loaded Amount, Brunauer–Emmett–Teller (BET) Specific Surface Area, and Absorption Amount

In the solid–liquid two phase catalytic reaction, the active site is on the intersurface of solid and liquid. Therefore, the specific surface area is very important for adsorption of the pollutants, which directly affects photocatalytic efficiency. The specific surface area of g-C_3_N_4_-TiO_2_ hybrids are investigated with N_2_ adsorption and desorption isotherms, and estimated by BET method. As shown in Table 1, combined with TiO_2_, the specific surface area is largely improved. However, the different loaded amount of g-C_3_N_4_ shows little effect on the specific surface area of g-C_3_N_4_-TiO_2_, which changed from 112.98 to 120.85 m^2^/g. In addition, the adsorption experiments matched the value of the specific surface area of the samples well.

**Figure 6:**
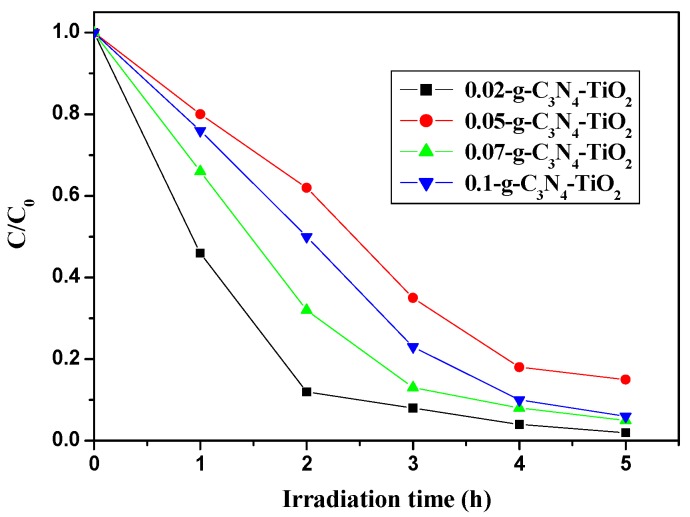
Temporal curves of the photodegradation of BDE209 with *x*-g-C_3_N_4_-TiO_2_ (*x* = 0.02 to 0.1) under visible irradiation. BDE209: 1.0 × 10^−5^ mol/L; g-C_3_N_4_-TiO_2_: 1 mg/mL; solvent: CH_3_OH; wavelength >420 nm; anoxic condition.

Photo-reductive activity of *x*-C_3_N_4_-TiO_2_ hybrids (*x* = 0.02, 0.05, 0.07, 0.1) was investigated by degradation of BDE209 under visible light irradiation. As shown in Figure 6, all samples of g-C_3_N_4_-TiO_2_ display efficient degradation of BDE209. The photoreductive efficiencies of g-C_3_N_4_-TiO_2_ hybrids can not are not match with the increase of the loaded amount of g-C_3_N_4_ from 0.05 to 0.1. 0.02-g-C_3_N_4_-TiO_2_ shows the highest reaction rate among all the g-C_3_N_4_-TiO_2_ hybrids. The degradation rates fit with pseudo-first-order model kinetics. With the loaded mass percentage of g-C_3_N_4_ changing from 0.05 to 0.1, the reaction rates show a certain degree of decrease compared with 0.02-g-C_3_N_4_-TiO_2_. The optimum loaded amount of C_3_N_4_ in the hybrids is 2 wt %.

#### 2.2.3. Photostabilty of g-C_3_N_4_-TiO_2_

**Figure 7:**
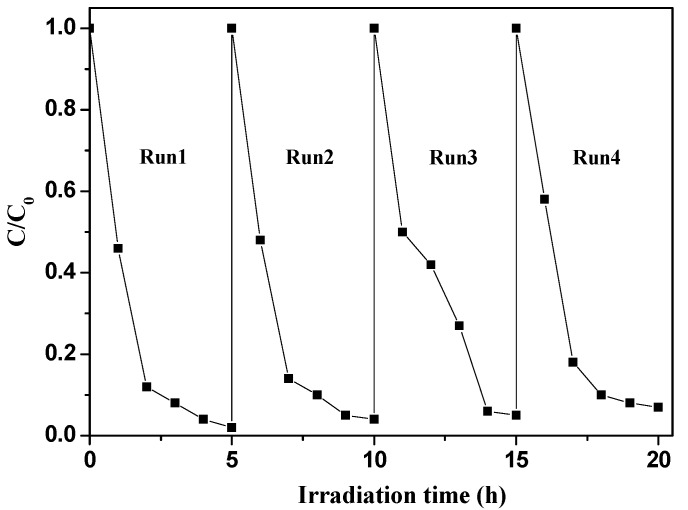
Cycling runs in the degradation of BDE209 with 0.02-g-C_3_N_4_-TiO_2_ under visible irradiation. BDE209: 1.0 × 10^−5^ mol/L; 0.02-g-C_3_N_4_-TiO_2_: 1 mg/mL; solvent: CH_3_OH; wavelength >420 nm; anoxic condition.

Photostability is the key factor for a catalyst in practical application. So, recycling experiments were performed to test the photostability of g-C_3_N_4_-TiO_2_. As shown in Figure 7, after four cycles, the photocatalyst still has strong photo-reductive ability for the degradation of BDE209. The reaction rates show no obvious decrease. This implies that g-C_3_N_4_-TiO_2_ shows an excellent heterojunction photocatalytic activity. In the heterojunction g-C_3_N_4_-TiO_2_, the recombination of electrons and holes are largely inhibited, and the separation of electrons and holes is obviously enhanced. Therefore, g-C_3_N_4_-TiO_2_ exhibits not only excellent photoreductive activity, but also good photostability for the degradation of BDE209.

#### 2.2.4. The Effect of Trapping Agents

**Figure 8:**
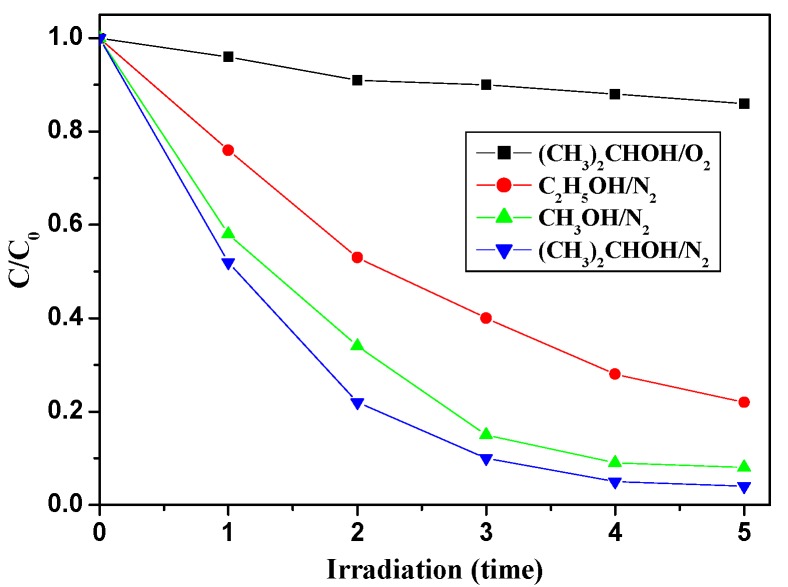
Effect of hole trapping agent on the photoreductive degradation of BDE209 by g-C_3_N_4_-TiO_2_ under visible light irradiation. BDE209: 1.0 × 10^−5^ mol/L; 0.02-g-C_3_N_4_-TiO_2_: 1 mg/1 mL; wavelength >420 nm; solvent: CH_3_CN; trapping agents: isopropanol, methanol, ethanol 0.1 mL.

Hole trapping agent experiments are performed to declare the mechanism of the reaction. As seen from Figure 8, the reaction rate is fastest in the presence of isopropyl alcohol, and the slowest rateis using ethanol as a scavenging agent. In the trapping experiment, the degradation rate of BDE209 changes by different trapping agents.

## 3. Discussion

The samples of g-C_3_N_4_-TiO_2_ show highly improved photoreductive capability for the degradation of polybromodiphenyl ethers compared with g-C_3_N_4_ under visible light irradiation. This implies that the well-matched band gap in the heterojunction g-C_3_N_4_-TiO_2_ greatly improves the photoreductive efficiency. 0.02-g-C_3_N_4_-TiO_2_ shows the highest reaction rate among all the g-C_3_N_4_-TiO_2_ hybrids. This indicates that a small amount of C_3_N_4_ (mass percentage is wt %) in C_3_N_4_-TiO_2_ hybrids may highly improve the photocatalytic activity; on the contrary, a greater loaded amount of C_3_N_4_ will inhibit the degradation of BDE209.

**Figure 9:**
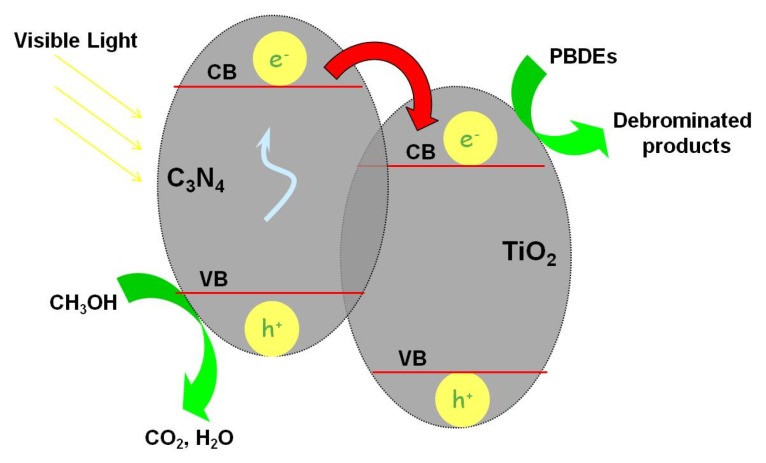
Proposed mechanism of the degradation of PBDEs with g-C_3_N_4_-TiO_2_ in CH_3_OH solution under visible irradiation. CB: conduction band; VB: valence band.

The hole trapping agent experiments have been performed to declare the rate-determining step and the mechanism of the reaction. During the photocatalytic reaction, an electron is excited from VB into CB of C_3_N_4_ under visible light irradiation, which results in the generation of VB hole and CB electron. The VB hole is scavenged by hole trapping agent, and the electron is accumulated in CB of C_3_N_4_. Under the anaerobic photocatalytic conditions, the possible reductive species that are responsible for the reduction of BDE209 are CB electrons. In the hole trapping agent experiment, the reaction rates are in the order of *i*-PrOH > MeOH > EtOH, which is in accordance with the order of the oxidation ability of alcohols with the VB hole via dehydrogenation. The oxidation of alcohols by the VB hole directly results in the accumulation of the electron in CB. This indicates that the key factor of the degradation reaction is the rate of electron accumulation in CB, which is the rate-determining step in the photoreductive degradation of BDE209 by g-C_3_N_4_-TiO_2_. In order to prove the key role of CB electrons in the photocatalytic degradation of BDE209, another control experiment has been performed. This control experiment is conducted under the same conditions with isopropyl alcohol as trapping agent, but O_2_ is purged into the reaction solution. Under visible light irradiation, the degradation of BDE209 is largely depressed in the O_2_-purged condition. O_2_ can react quickly with the CB electron, which results in the inhibition of the degradation of BDE209. This result again proves that it is the CB electron that makes BDE209 reductively degrade and that the rate of accumulation of CB electrons is the rate-determining step. Based on the above experiments, a possible reaction mechanism has been proposed with the degradation of BDE209 in CH_3_OH solution by C_3_N_4_-TiO_2_ (Figure 9). Under visible light irradiation, C_3_N_4_ is excited and formsVB hole and CB electron. When the VB holes are scavenged by trapping agents,the electrons will accumulate in CB. In theheterojunction g-C_3_N_4_-TiO_2_, the CB of C_3_N_4_ (−1.30 eV) is more negative than that of TiO_2_ (−0.25 eV). So, the electron in CB of C_3_N_4_ prefers to transfer to the CB of TiO_2_ *via* the interface of heterojunctiong- C_3_N_4_-TiO_2_. BDE209 receives the electrons in CB of TiO_2_ and forms C_10_Br_9_O∙and Br^−^. The C_10_Br_9_O radical abstracts a hydrogen atom from methanol (hole trapping agent) and yields lower bromo congeners C_10_Br_9_OH, and CH_3_OH transforms into HCHO by losing a hydrogen atom.

## 4. Materials and Methods

### 4.1. Materials

BDE209 was purchased from Sigma-Aldrich (St. Louis, MO, USA). BDE203, BDE204, and a standard solution of PBDEs (EO5103) were purchased from Cambridge Isotope Laboratories (CIL, Andover, MA, USA). Tetra-n-butyl titanate, melamine, isopropanol, methanol, ethanol, and acetonitrile were analytical reagents (Chemical Co., Shanghai, China). They were used without further purification. Deionized and doubly distilled water was used throughout the study.

### 4.2. Methods

#### 4.2.1. Synthesis of g-C_3_N_4_-TiO_2_

A certain amount of melamine was added into 100 mL isopropanol solution and stirred at ambient temperature for 2 h prior to addition of 4 mL Ti(OBu)_4_. After stirred 3 h, 40 mL deionized water was added dropwise into the suspension. The mixture was put into a drying oven for 24 h at 60 °C and powder was obtained, which was collected and annealed at 500 °C for 4 h to get the g-C_3_N_4_-TiO_2._ By adjusting the mass percentage ratio of g-C_3_N_4_ to TiO_2_, different amounts of g-C_3_N_4_ were combined with TiO_2_, obtained *x*-g-C_3_N_4_-TiO_2._ (*x* = 0, 0.02, 0.05, 0.07, 0.1; *x* refers to the mass percentage of g-C_3_N_4_ in g-C_3_N_4_-TiO_2_).

#### 4.2.2. Characterization

The morphology of the catalyst samples were determined by scanning electron microscope (SEM) on a Hitachi S4300 and transmission electron microscope (TEM) (Philips CM200 FEG TEM at 200 kV, Tokyo, Japan). The X-ray diffraction patterns of the catalyst powders were performed on a Rigaku D/Max-2500 diffractometer with the Cu Kα radiation (1.5406 Å). The X-ray photoelectron spectroscopy (XPS) measurements were carried using a ESCA lab 220i-XL spectrometer with Al Kα (1486.6 eV) X-ray source and a charge neutralizer. The specific surface areas of different C_3_N_4_-TiO_2_ samples were determined by Brunauer–Emmett–Teller (BET) method. BDE209 was quantified with a SHIMADZU HPLC system (LC-20AT pump and UV/VIS SPD-20A detector, Kyoto, Japan) with a DIKMA Platisil ODS C-18 column (250 × 4.6 mm, 5 μm film thickness). The mobile phase was 98% acetonitrile and 2% water at 1 mL/min, and the detector wavelength was set at 240 nm. The quantification of BDE209 was performed by a calibration curve with a BDE-209 standard. Products were detected by GC-μECD analysis with GC (Agilent 7890A, Santa Clara, CA, USA) equipped with an electron capture detector (ECD) (Agilent Technologies Co., Santa Clara, CA, USA), a programmable pressure on-column injection port, and a DB-5 capillary column (30 m × 50 μm, i.d. × 0.1 μm film thickness). Splitless 10 μL injection was performed manually at 300 °C. The carrier gas was helium at a constant flow rate of 1.0 mL/min. The oven temperature was kept at 100 °C for 2 min, increased at 15 °C/min to 230 °C, then increased at 5 °C/min to 270 °C, and finally increased at 10 °C/min to 320 °C for 10 min. The standard samples of BDE203, BDE204, and PBDEs (EO5113) were used to identify the degradation products. At given times, 1 mL aliquots were collected and centrifuged, and filtered to remove the solid catalyst particles before analysis with HPLC and GC-μECD.

#### 4.2.3. Experimental Setup

BDE209 stock solution (1 × 10^−3^ mol/L) in tetrahydrofuran was diluted with methanol (1 × 10^−5^ mol/L). Ten milligrams of g-C_3_N_4_-TiO_2_ was added to 10 mL BDE209 solution in a Pyrex vessel. Reaction solution was magnetically stirred during the irradiation. The Pyrex vessel was purged with argon for 15 min to remove O_2_ and protected under argon atmosphere during the irradiation. A PLS-SXE300 Xe lamp (Beijing Trusttech Co. Ltd., Beijing, China) was used as the light source. A cutoff filter (λ > 420 nm) was placed to ensure visible light irradiation. To investigate the effect of trapping agent on the reaction kinetics, a given amount of isopropanol, ethanol, and methanol were added into BDE209 acetonitrile solution under identical conditions.

## 5. Conclusions

The photocatalysts of g-C_3_N_4_-TiO_2_ synthesized by simple one-pot thermal transformation method show highly improved photoreductive activity for the degradation of BDE209 under visible light irradiation. Among all the hybrids, 0.02-C_3_N_4_-TiO_2_ with 2 wt % TiO_2_ loaded showed the highest reaction rate—15 times higher than bare g-C_3_N_4_. The heterojunction of g-C_3_N_4_-TiO_2_ with well-matched band gaps strengthened the absorption intensity of TiO_2_ and g-C_3_N_4_, and contributed more effective charge carrier separation. Therefore, the heterojunction of g-C_3_N_4_-TiO_2_ showed highly enhanced photoreductive performance. The trapping experiment indicated that the rate of electron accumulation in the conductive band is the rate-determining step in the degradation reaction.

## Figures and Tables

**Table 1:** Brunauer–Emmett–Teller specific surface area (*S*_BET_, m^2^/g), absorption amount, and estimated pseudo-first-order kinetic constant (*k*) for the degradation of BDE209.

Catalyst	Mass Percentage of C_3_N_4_ in C_3_N_4_-TiO_2_	*S*_BET_ (m^2^/g)	Absorption Amount	Kinetic Constant *k* (h^−1^)
g-C_3_N_4_	0	27.84	0.17	0.05
0.02-g-C_3_N_4_-TiO_2_	0.02	120.85	0.27	0.78
0.05-g-C_3_N_4_-TiO_2_	0.05	115.16	0.24	0.42
0.07-g-C_3_N_4_-TiO_2_	0.07	114.18	0.24	0.63
0.1-g-C_3_N_4_-TiO_2_	0.1	112.98	0.22	0.60

## Supplementary Materials

The following are available online at www.mdpi.com/2079-4991/7/4/76/s1.
