# Supplementary material for: Highly Enhanced Photoreductive Degradation of Polybromodiphenyl Ethers with g-C3N4/TiO2 under Visible Light Irradiation
Source: Nanomaterials (Basel). 2017 Apr 3;7(4):76. doi: 10.3390/nano7040076 (PMC5408168; doi:10.3390/nano7040076)
Supplement: Supplementary file 1 [file nanomaterials-07-00076-s001.pdf]

Highly Enhanced Photoreductive Degradation of Polybromodiphenyl Ethers with g-C_3N_4/TiO_2 under Visible Light Irradiation

Weidong Ye [1], Yingying Shao [1], Xuefeng Hu [2], Chulin Liu [1] and Chunyan Sun [1,*]

[1] Department of Chemistry, Shaoxing University, Shaoxing 312000, Zhejiang, China; zjpjyeweidong@163.com (W.Y.); hmilyariel@icloud.com (Y.S.); liuchunlin@usx.edu.cn (C.L.)

[2] Key laboratory of Coastal Zone Envirenmental Process and Ecological Remediation, Yantai Institute of Coastal Zone Research, Chinese Academy of Sciences, Yantai 264003, Shandong, China; xfhu@yic.ac.cn

[*] Correspondence: chunyansun74@gmail.com; Tel./Fax: 86-575-8834-1521

Supporting Information

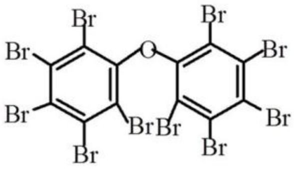

Figure S1. The structure of BDE209

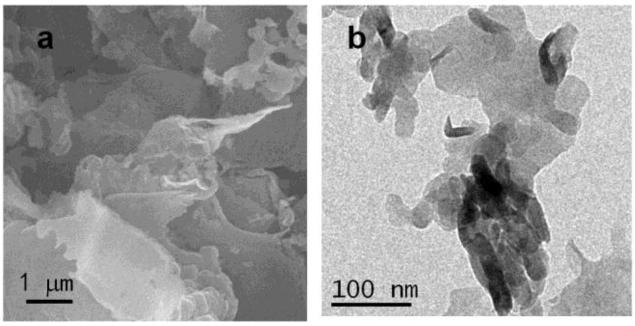

Figure S2 (**a**) SEM image of g-C_3N_4; (**b**) TEM image of g-C_3N_4

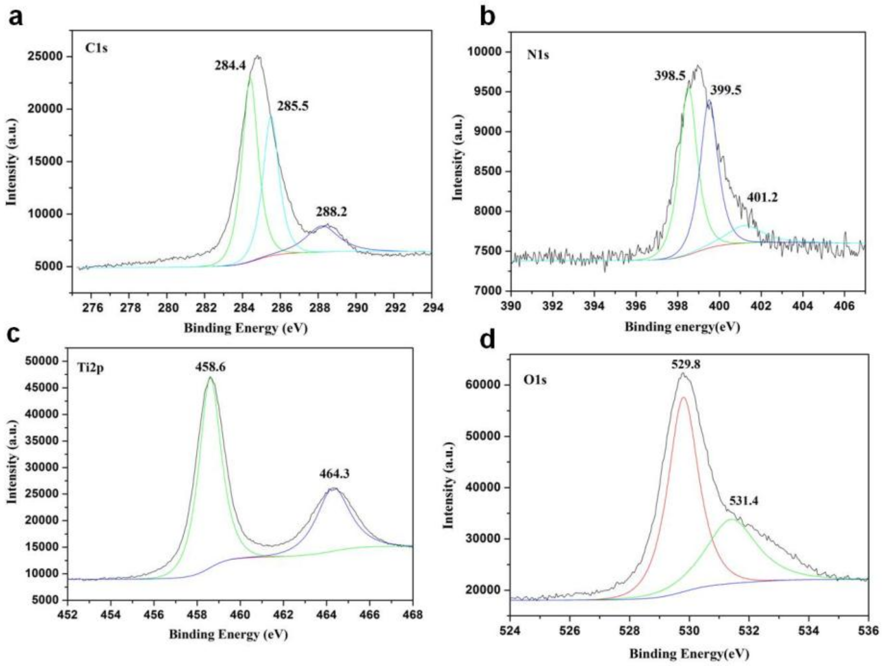

Figure S3. XPS spectra of C1s (**a**) ,N1s (**b**),Ti2p (**c**) and O1s (**d**) for 0.02-C_3N_4-TiO_2

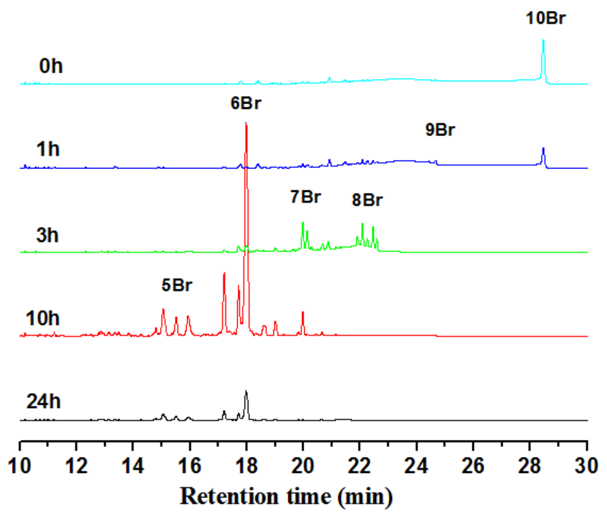

Figure S4 GC-μECD chromatograms of degradation products of BDE209 by 0.02-g-C_3N_4-TiO_2 in different irradiation times.
